# 三相中空纤维液相微萃取-高效液相色谱法测定铁皮石斛和金线莲中3种植物生长调节剂

**DOI:** 10.3724/SP.J.1123.2023.03007

**Published:** 2023-08-08

**Authors:** Pingping WU, Renyi LIN, Liying HUANG

**Affiliations:** 福建医科大学药学院,福建 福州 350122; School of Pharmacy, Fujian Medical University, Fuzhou 350122, China

**Keywords:** 三相中空纤维液相微萃取, 高效液相色谱, 植物生长调节剂, 铁皮石斛, three-phase hollow fiber liquid phase microextraction (3P-HF-LPME), high performance liquid chromatography (HPLC), plant growth regulators (PGRs), *Dendrobium officinale*

## Abstract

在铁皮石斛和金线莲药材的人工培育过程中通常需要使用各种植物生长调节剂(PGRs),然而使用过量的PGRs不仅会影响药材的品质和疗效,而且会引发一系列的安全性问题,因此建立精准分析测定药材中残留的痕量PGRs技术方法具有重要意义。本研究基于三相中空纤维液相微萃取(3P-HF-LPME)-高效液相色谱法,建立了一种同时测定铁皮石斛和金线莲药材中吲哚乙酸、吲哚丁酸、萘乙酸3种PGRs的分析方法。首先通过参数优化确定最佳色谱条件,然后采用超声+低温静置的提取手段制备样本溶液,优化影响3P-HF-LPME萃取效果的各个参数。确定的最佳前处理过程如下:以正辛醇作为中间相(萃取剂), pH 11.0的氢氧化钠溶液作为内相,稀盐酸调节外相溶液pH值为3.0,并在外相溶液中添加质量浓度为150 g/L的氯化钠溶液,在温度为40 ℃、搅拌速度为1600 r/min条件下萃取2.0 h。3种PGRs采用Welch Ultimate XB-C_18_色谱柱(250 mm×4.6 mm, 5 μm)进行分离,以乙酸水溶液-甲醇(45∶55, v/v)为流动相进行等度洗脱。实验结果表明,在优化的条件下,所测定的3种PGRs在0.5~100.0 μg/L范围内线性关系良好(决定系数(*r*^2^)=0.9999),检出限(LOD)为0.02~0.15 μg/L,加标回收率为88.5%~102.2%,相对标准偏差(RSD)≤3.7%(*n*=3)。该方法对铁皮石斛和金线莲药材中3种PGRs的萃取效率为42.0%~86.8%,富集倍数为140~289。该方法灵敏、准确、可靠、环保,且富集倍数高,适用于铁皮石斛和金线莲药材中残留的微量酸性PGRs的检测。

## Abstract (en)

*Dendrobium officinale* (*D. officinale*) and *Anoectochilus roxburghii* (*A. roxburghii*) are precious raw materials for traditional Chinese medicine. The growing demand for *D. officinale* and *A. roxburghii* cannot be met by current production techniques. Hence, the widespread artificial cultivation of *D. officinale* and *A. roxburghii* using substantial amounts of plant growth regulators (PGRs) has emerged. The excessive use of PGRs not only affects the quality and efficacy of medicinal materials but also causes a series of safety issues. Therefore, expanding research on residual PGRs in valuable Chinese medicinal materials is important to avoid the health hazards caused by these substances. Unfortunately, the identification of PGRs is challenging because of their trace and complex matrices.

High performance liquid chromatography (HPLC) has become one of the mainstream analytical methods for PGR determination. An important consideration in the application of this technique to the detection of trace acidic PGRs is how to improve its accuracy and sensitivity. Three-phase hollow fiber liquid phase microextraction (3P-HF-LPME) has the advantages of a high enrichment factor, complex sample purification ability, low reagent consumption, low cost, and easy integration with chromatographic systems. Thus, the 3P-HF-LPME method overcomes the many shortcomings of traditional sample pretreatment methods. In this study, a novel, simple, and effective analytical method based on 3P-HF-LPME combined with HPLC was developed to extract, purify, enrich, and detect three trace acidic PGRs (indole-3-acetic acid, naphthyl acetic acid and indolebutyric acid) in *D. officinale* and *A. roxburghii*. The chromatographic separation conditions and 3P-HF-LPME model parameters were systematically optimized for this purpose. First, the sample solution was prepared by ultrasonication and low-temperature standing, and then adjusted to pH 3.0 using dilute hydrochloric acid. The sample solution (10 mL) and NaCl (1.50 g) were stored in a 15 mL brown extraction bottle with a built-in magnetic stirrer. Next, 30 μL of NaOH solution (pH 11.0) as the inner phase solution was injected into the inner cavity of a hollow fiber tube, which was subsequently sealed at both ends. The hollow fiber tube was soaked in *n*-octanol for 5 min and dried naturally to remove excess extraction solvent from its surface. Finally, the fiber tube was placed in a brown extraction bottle and stirred using a thermostatic magnetic stirrer at 40 ℃ and 1600 r/min for 2 h. After extraction, the three target analytes were separated on a Welch Ultimate XB-C_18_ column (250 mm×4.6 mm, 5 μm) under isocratic elution conditions using acetic acid aqueous solution and methanol (45∶55, v/v) as the eluent. The results indicated that the three PGRs showed good linearity in the range of 0.5-100.0 μg/L (coefficients of determination (*r*^2^)=0.9999), with limits of detection (LODs) of 0.02-0.15 μg/L. The method recoveries were 88.5-102.2%, with relative standard deviations (RSDs) of less than 3.7% (*n*=3). The extraction efficiencies and enrichment factors of the three PGRs in 15 batches of fresh *D. officinale* and *A. roxburghii* products were found to be 42.0%-86.8% and 140-289. Full-scan mass spectrometry was used to further identify positive samples to avoid false-positive results and enhance the reliability of the experimental method. In summary, the proposed method is sensitive, accurate, reliable, environment friendly, and capable of high enrichment. It could be used to determine the residues of three acidic PGRs in *D. officinale* and *A. roxburghii*. Moreover, it can provide technical support for the residue detection of PGRs in other Chinese medicinal materials.

植物生长调节剂(plant growth regulators, PGRs)对植物生长、发育及衰老等方面起着重要的作用,通过人工添加PGRs,可以增加植物次生代谢物的累积量,改善品质、增加有效成分含量^[1⇓-3]^。因此,越来越多的PGRs应用于中药材尤其是铁皮石斛(*Dendrobium officinale* (Wall.) ex Lindl., *D. officinale*)和金线莲(*Anoectochilus roxburghii* (Wall.) Lindl., *A. roxburghii*)这些名贵药材的人工栽培过程中。据文献报道^[4,5]^,在铁皮石斛和金线莲药材栽培过程中通常会在基础培养基中加入吲哚乙酸(indole-3-acetic acid, IAA)、萘乙酸(naphthyl acetic acid, NAA)或者吲哚丁酸(indolebutyric acid, IBA)。然而使用过量的PGRs不仅会影响药材的品质和疗效,而且会引发一系列的安全性问题^[6⇓-8]^,因此,为避免过量残留的PGRs对人体造成健康隐患,建立精确测定残留量的技术方法,完善中药材中PGRs残留量的限量标准是非常重要的。

目前,应用于中药材中PGRs残留量检测的前处理技术主要有固相萃取(solid phase extraction, SPE)^[9⇓⇓-12]^、液液萃取(liquid liquid extraction, LLE)^[13⇓-15]^和各种新型微萃取技术^[16⇓⇓⇓-20]^。液液微萃取法(liquid liquid microextraction, LLME)^[21]^是在传统的LLE基础上发展的一种新型微萃取技术,克服了传统LLE消耗大量溶剂以及经典的SPE小柱操作繁琐且易堵塞等不足。尤其是中空纤维膜的引入使得LLME稳定性得到更大提升,在应用方面更为简便快速。三相中空纤维液相微萃取(3P-HF-LPME)具有富集倍数(EF)高、消耗试剂较少、使用成本低、易与色谱系统联用等优点,克服了传统方法的诸多不足,已用于环境、生物样品、药品等领域复杂样品中痕量成分的测定^[22]^。

近年来色谱技术的飞速发展使其在植物生长调节剂的检测领域发挥了重要作用。高效液相色谱法(HPLC)无需昂贵和复杂的设备,又具备重现性好、准确可靠、通用性突出等优点^[23⇓-25]^,在中药材中PGRs残留量的测定中应用较广。本工作建立了基于3P-HF-LPME结合HPLC检测铁皮石斛和金线莲中3种植物生长调节剂的方法,通过建立、优化3P-HF-LPME萃取模型和条件,实现了在最优条件下对3种目标分析物的净化富集,降低了基质效应;为防止假阳性检测结果,采用质谱的全扫描模式对阳性样品作进一步的鉴定,以增强实验结果的可信度。

## 1 实验部分

### 1.1 仪器、试剂与材料

LC-15C高效液相色谱仪(日本岛津公司); Agilent 6410BA型三重四极杆质谱仪(美国安捷伦公司); RE-2000旋转蒸发仪(上海亚荣生化仪器厂); DF-101S集热式恒温加热磁力搅拌器(湖南玉华仪器有限公司); IKA VORTEX GENIUS-3涡旋混合器(德国IKA仪器公司); UB-7型pH计(丹佛仪器公司); KQ-100TDV型高频数控超声波清洗器(江苏昆山市超声仪器有限公司); Neofuge18R高速冷冻离心机(香港力康发展有限公司)。S6/2型聚丙烯中空纤维(直径1800 μm,壁厚450 μm,表面孔径0.2 μm)购于北京北研众科科技有限公司。

正己醇、正庚醇、正辛醇、正癸醇、乙酸、IAA(纯度99%)、IBA(纯度99.5%)均购自上海阿拉丁生化科技股份有限公司;NAA(纯度98%)购自上海源叶生物科技有限公司;甲醇(色谱纯)购自上海Sigma有限公司;氯化钠(分析纯)购自广州西陇试剂有限公司;氢氧化钠(分析纯)购自上海泰坦科技股份有限公司。铁皮石斛、金线莲鲜品来源见表1,购买后均保存于4 ℃冰箱中。

**表1:** 铁皮石斛和金线莲样品信息

Name of medicinal material	Source	Nos.	Number of batches
*Dendrobium officinale* (Wall.) ex Lindl.	Fujian Yonggeng Agricultural Development Co., Ltd.	A	1
(*D. officinale*)	Fuqing Jiajia Agricultural Development Co., Ltd.	B	1
	Fuzhou Youye Agricultural Development Co., Ltd.	C1-C6	6
	Fujian Kanglin Agricultural Development Co., Ltd.	D1, D2	2
*Anoectochilus roxburghii* (Wall.) Lindl.	Fuzhou Youye Agricultural Development Co., Ltd.	E1, E2	2
(*A. roxburghii*)	Fujian Kanglin Agricultural Development Co., Ltd.	F1-F3	3

### 1.2 溶液配制

混合标准储备液:准确称取IAA、IBA、NAA标准品各10.0 mg于10 mL容量瓶中,加入甲醇溶解定容至刻度,配制成1.0 g/L的混合标准储备液,于4 ℃保存。

混合标准溶液系列:移取适量的混合标准储备液,用甲醇逐级稀释,配制质量浓度为100、50、10、5、1、0.5 μg/L的混合标准溶液系列。

样品溶液制备:称取烘干后的铁皮石斛或金线莲药材2.0 g,置于用锡纸包裹遮光的50 mL微量离心管中并加入30 mL甲醇,用匀浆机粉碎样品。将粉碎后的样品匀浆液超声30 min后,放置于4 ℃冰箱中浸提12 h。随后于35 ℃、 9000 r/min条件下离心15 min,吸取上层清液,向下层样品残渣中重新加入30 mL甲醇。重复上述操作2次,合并3次上清液并将其转移置于250 mL棕色梨形蒸发瓶中,在旋转蒸发仪上蒸发近干(温度为40 ℃),加入1 mL甲醇复溶,并加水定容至100 mL的棕色容量瓶中,用稀盐酸溶液调节pH至3.0,完成样品溶液的制备。

### 1.3 色谱条件

色谱柱:Welch Ultimate XB-C_18_ (250 mm×4.6 mm, 5 μm);流动相组成:乙酸水溶液(pH 3.0)-甲醇(45∶55, v/v);等度洗脱;流速:1 mL/min;进样量:20 μL;检测波长:280 nm;柱温:30 ℃。

### 1.4 质谱条件

离子源:电喷雾离子源(ESI源);模式:正离子模式;扫描范围:*m/z* 50~400;扫描速度:358 u/s;接口电压:4.5 kV;加热块、接口和去溶剂化温度分别为250、350和250 ℃;雾化气和干燥气为氮气,流速分别为1.5 L/min和12 L/min。

### 1.5 3P-HF-LPME

中空纤维管使用前先剪成2 cm的小段,并用甲醇超声清洗10 min,经烘箱干燥后,用封口机将其中一端封口。取10 mL样品溶液或一定浓度的混合标准溶液置于15 mL棕色萃取瓶中(内置磁力搅拌子),加入1.50 g NaCl。用50 μL微量进样器将30 μL内相溶液(pH 11.0的NaOH溶液)注入中空纤维管内腔并封口。将两端封口的中空纤维管置于萃取剂正辛醇(中间相)中浸泡5 min,取出自然晾干以除去表面多余的萃取剂。将纤维管放入棕色萃取瓶内,置于恒温磁力搅拌器中,保持温度40 ℃,以1600 r/min的搅拌速度萃取2.0 h。萃取结束后取出中空纤维管,一端剪开吸取内相溶液,记录吸取的体积,并用流动相稀释20倍。经滤膜过滤后,用HPLC对其进行分析。

## 2 结果与讨论

### 2.1 萃取工艺设计与优化

#### 2.1.1 萃取剂的优化

在3P-HF-LPME中,萃取剂对样品溶液中目标分析物的萃取效率起到了至关重要的作用,实验考察了正己醇、正庚醇、正辛醇、正癸醇等4种正构醇对目标分析物萃取效率的影响,正构醇中的-OH和目标分析物中的-COOH形成氢键,能够使目标分析物的溶解度增加,如图1所示,萃取效率开始随正构醇分子中C原子数增加而增加,正辛醇对3种组分的萃取效率最高,之后萃取效率随正构醇中C原子数增加而降低,表明正辛醇与3种组分形成的氢键能力最强,因此选择正辛醇作为萃取剂。

**图1:**
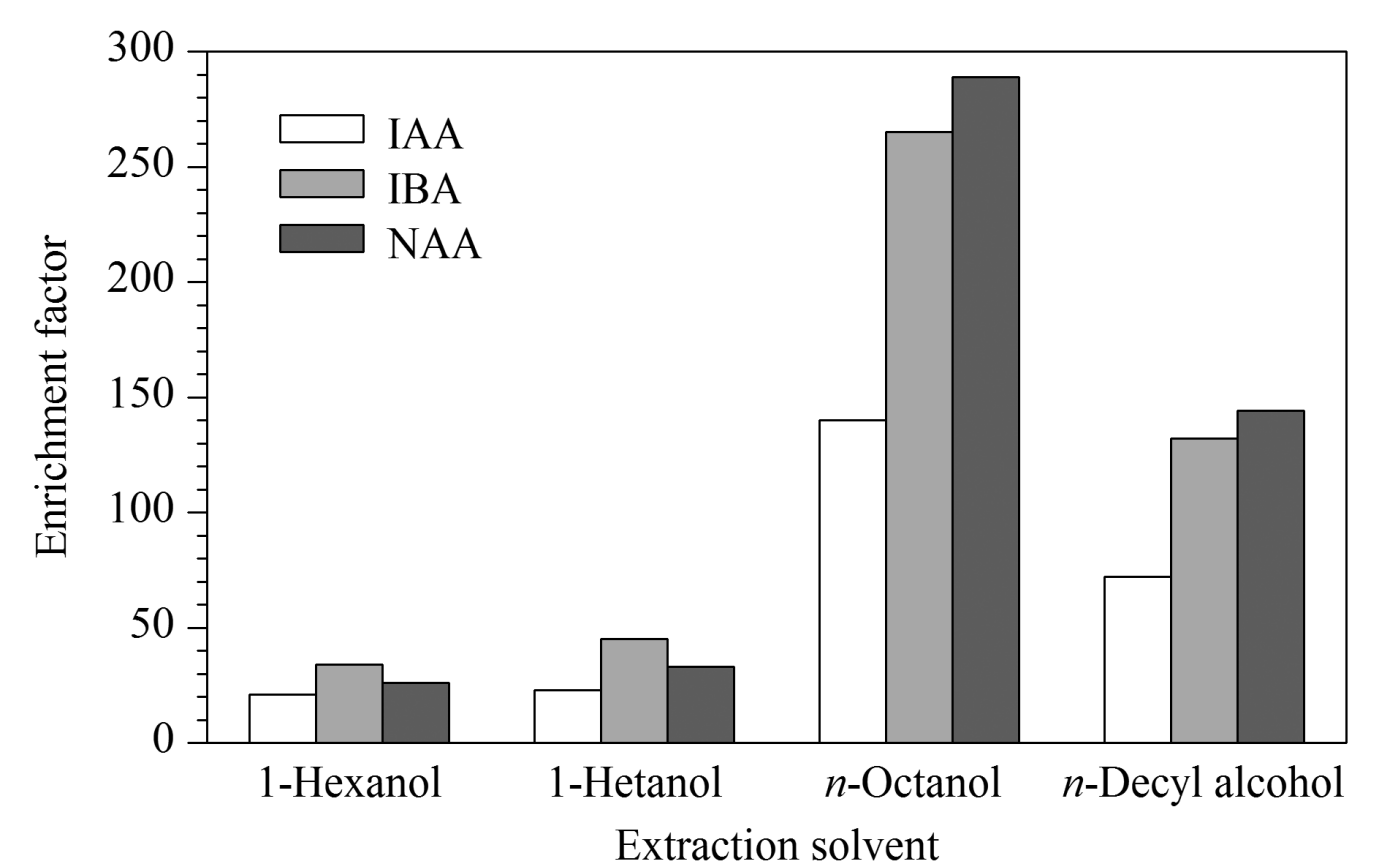
萃取剂对萃取效率的影响

#### 2.1.2 内外相pH的优化

IAA、IBA、NAA均为不易溶于水的弱酸性物质,在外相样品溶液中加入适量的酸有利于它们以分子状态存在,增加在有机相中的溶解度从而提高萃取效率,实验采用稀盐酸溶液调节外相pH值为2.5、3.0、3.5、4.0、4.5,考察其萃取效率,其结果如图2a所示,当溶液pH在3.5~4.5时,目标分析物存在部分解离,不能有效地被中间相萃取,故选择调节外相pH为3.0作为最佳条件。当在内相中添加适量的碱使目标分析物呈离解状态,即能使分析物迅速从有机溶剂中反萃出来,又能够防止其重新扩散回有机溶剂中,实验采用NaOH溶液调节内相pH值为9.0~13.0,考察其萃取效率,其结果见图2b,随着pH值的升高,3种目标分析物的萃取量均增加,pH在11.0~12.0时基本达到最大萃取量,故调节内相pH为11.0作为最佳条件。

**图2:**
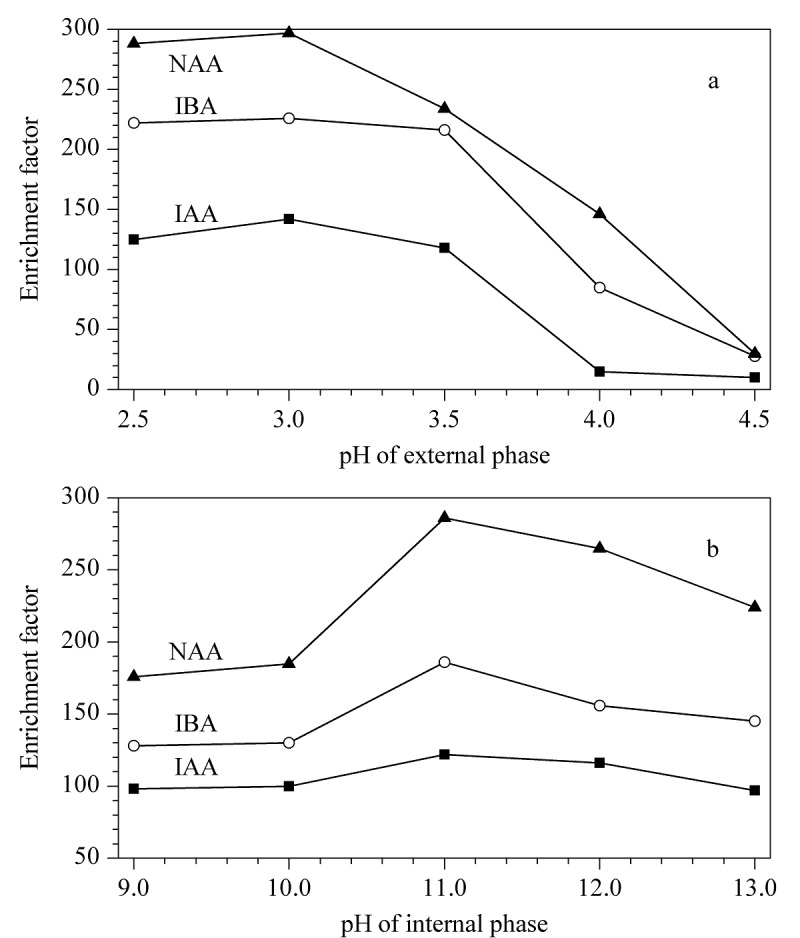
内外相pH值对萃取效率的影响

#### 2.1.3 氯化钠添加量的优化

实验考察了样品溶液中NaCl的添加量(0~250 g/L)对目标分析物萃取效率的影响,结果如图3所示,在NaCl质量浓度为0~150 g/L范围内,随着NaCl添加量的增加,盐析效应使目标分析物在外相中溶解度降低,促进其在中间相萃取,萃取效率也随之增加;当NaCl质量浓度超过150 g/L时,目标分析物的萃取量反而减少,这可能是因为样品溶液的黏度变大,导致其在两相之间传质能力降低,从而降低了萃取效率。因此,最终样品溶液中NaCl的添加量为150 g/L。

**图3:**
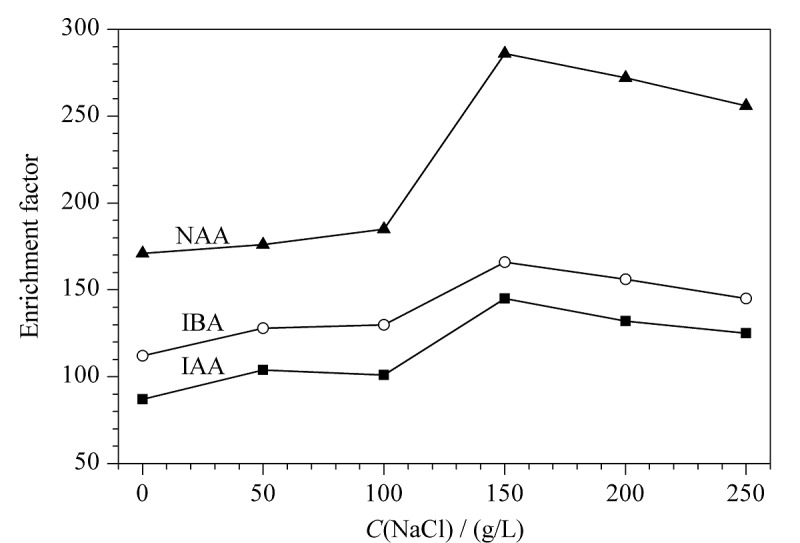
不同盐添加量对萃取效率的影响

#### 2.1.4 搅拌速度、温度、时间的优化

3P-HF-LPME是一个动态传质的平衡过程,温度的升高、搅拌速度的加快均会增强目标分析物在三相中的传质速率。考察了搅拌速度(400、800、1200、1600和1800 r/min)、萃取时间(0.5、1.0、1.5、2.0、2.5、3.0 h)、萃取温度(20、25、30、35、40、45 ℃)对萃取效果的影响,结果表明,当搅拌速度、萃取时间、萃取温度分别为1600 r/min、40 ℃、2.0 h时,萃取效果最佳。

### 2.2 方法学考察

#### 2.2.1 线性范围、检出限、定量限和富集倍数

考察了方法的线性范围、检出限、定量限和富集倍数,实验结果见表2。在优化条件下,分析不同质量浓度的混合标准溶液系列,以标准溶液质量浓度(*X*, μg/L)为横坐标,峰面积(*Y*)为纵坐标进行线性回归,3种PGRs在0.5~100.0 μg/L内线性良好,决定系数(*r*^2^)等于0.9999。按照3倍信噪比和10倍信噪比确定化合物的检出限(LOD)和定量限(LOQ), 3种PGRs的检出限为0.02~0.15 μg/L,定量限为0.06~0.50 μg/L。富集倍数按照公式EF=*C*_i_/*C*_e_计算,*C*_i_(μg/L)表示萃取后内相中目标分析物的质量浓度;*C*_e_(μg/L)表示萃取前目标分析物的质量浓度,3种PGRs的富集倍数为140~289。

**表2:** 3种PGRs的回归方程、线性范围、检出限、定量限和富集倍数

Analyte	Regression equation	*r*^2^	Linear range/(μg/L)	LOD/(μg/L)	LOQ/(μg/L)	EF
IAA	*Y*=0.00580*X*-0.00030	0.9999	0.5-100.0	0.15	0.50	140
IBA	*Y*=0.00930*X*-0.00080	0.9999	0.5-100.0	0.10	0.33	267
NAA	*Y*=0.01360*X*+0.00200	0.9999	0.5-100.0	0.02	0.06	289

PGRs: plant growth regulators. *Y*: peak area; *X*: mass concentration; μg/L. *r*^2^: coefficient of determination.

#### 2.2.2 精密度

为考察检测方法的精密度,对100 μg/L的混合标准溶液,按1.5节操作并进样分析,每日平行测定6次,分析并计算相对标准偏差(RSD)作为日内精密度。连续3日重复上述操作,其结果的RSD值作为日间精密度。结果表明,测定IAA、IBA和NAA 3种PGRs的日内精密度分别为5.0%、1.6%和2.7%,日间精密度为6.0%、4.7%和4.9%。

#### 2.2.3 加标回收率

为了评估检测方法的准确性,选择检测结果为阴性的铁皮石斛样品溶液为空白基质,分别在低(20 μg/L, 1.0 μg/g)、中(40 μg/L, 2.0 μg/g)、高(80 μg/L, 4.0 μg/g)3种不同加标水平下添加混合标准溶液,按照优化的分析方法进行分析测定,每个水平平行操作3次,计算加标回收率。结果表明,3种PGRs的平均回收率为88.5%~102.2%,RSD为1.3%~3.7%(见表3)。

**表3:** 3种PGRs在铁皮石斛样品中的加标回收率及其RSD(*n*=3)

Analyte	Added/(μg/g)	Found/(μg/g)	Recovery/%	RSD/%
IAA	1.0	1.02	101.4	2.0
	2.0	2.04	102.2	2.9
	4.0	3.92	98.3	2.4
IBA	1.0	0.91	90.5	3.7
	2.0	1.89	94.5	1.6
	4.0	3.54	88.5	2.1
NAA	1.0	0.92	92.4	1.3
	2.0	2.01	101.1	1.6
	4.0	3.75	93.8	2.0

### 2.3 样品分析

在优化后的实验条件下,对10批烘干后的铁皮石斛和5批金线莲药材进行3种PGRs含量检测,混合标准溶液(10 μg/L)、D1号样本溶液、空白加标样品溶液(40 μg/L)萃取前后的色谱图见图4。结果表明,标号为D1和D2的铁皮石斛中含有NAA,含量分别为53.7和82.8 ng/g,其他批次的样品均未检测到3种PGRs。

**图4:**
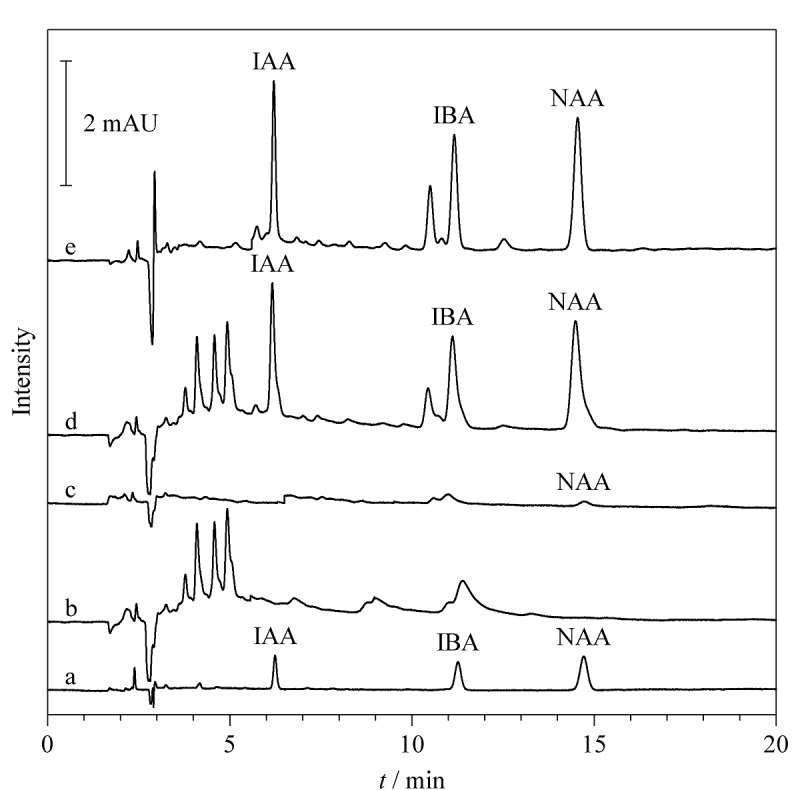
混合标准溶液、D1样品溶液及空白样品加标溶液萃取前后的色谱图

### 2.4 质谱分析

由于样品溶液基质较为复杂,可能存在基质干扰,因此阳性样品采用质谱分析进行确认。将NAA标准溶液和萃取后的D1、D2号铁皮石斛样品溶液按1.4节质谱条件检测分析,结果如图5所示,其中*m/z* 187.10为NAA的[M+H]^+^准分子离子峰,*m/z* 209.20为NAA的[M+Na]^+^准分子离子峰。综合色谱保留时间、一级质谱数据,可以确定D1、D2号铁皮石斛样品溶液中存在NAA。

**图5:**
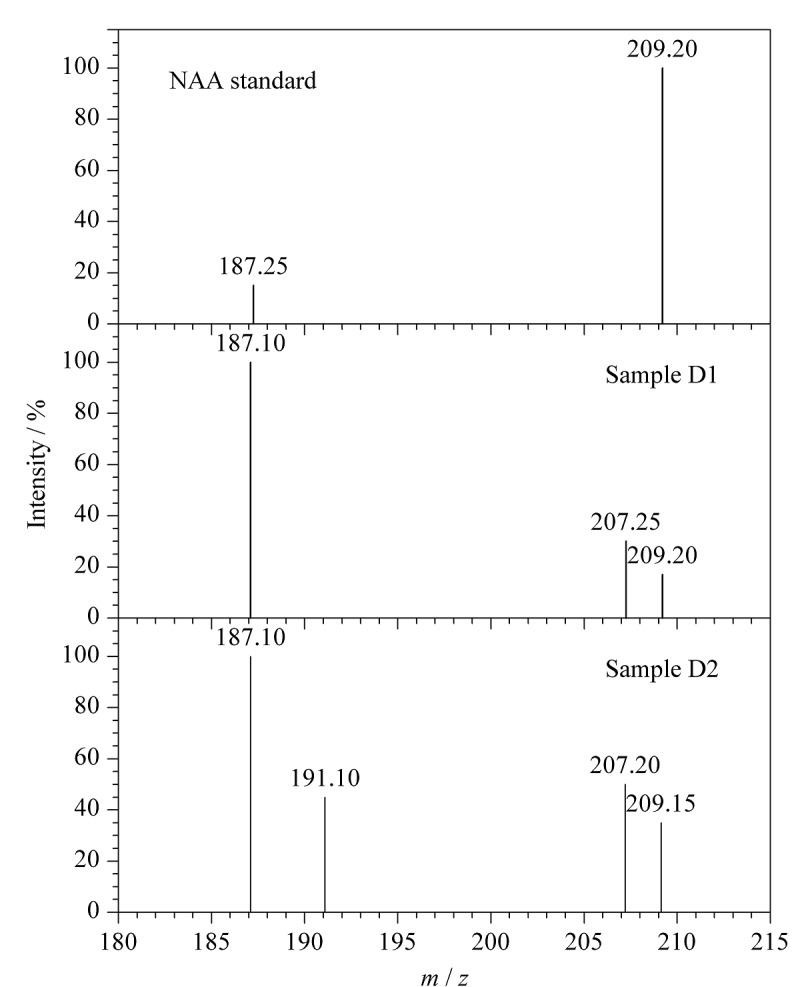
标准品与样本溶液萃取后的质谱图

## 3 结论

采用3P-HF-LPME新型样品前处理技术结合HPLC检测技术对铁皮石斛和金线莲中的3种植物生长调节剂残留进行富集和分离检测。实验结果表明该方法富集倍数高,同时具备较好的精密度和灵敏度,实验成本较低,对环境友好,可为中药材植物生长调节剂的残留检测提供技术支撑。
